# PET amyloid in normal aging: direct comparison of visual and automatic processing methods

**DOI:** 10.1038/s41598-020-73673-1

**Published:** 2020-10-07

**Authors:** Sven Haller, Marie-Louise Montandon, Johan Lilja, Cristelle Rodriguez, Valentina Garibotto, François R. Herrmann, Panteleimon Giannakopoulos

**Affiliations:** 1 CIRD Centre d’imagerie Rive Droite, Geneva, Switzerland; 2 Department of Surgical Sciences, Radiology, Uppsala University, Uppsala, Sweden; 3 Faculty of Medicine, University of Geneva, Geneva, Switzerland; 4 Department of Rehabilitation and Geriatrics, Geneva University Hospitals and University of Geneva, Geneva, Switzerland; 5 Department of Psychiatry, University of Geneva, Geneva, Switzerland; 6 Clinical Memory Research Unit, Department of Clinical Sciences, Lund University, Malmö, Sweden; 7 Hermes Medical Solutions, Stockholm, Sweden; 8 Division of Institutional Measures, Medical Direction, Geneva University Hospitals, Geneva, Switzerland; 9 Division of Nuclear Medicine and Molecular Imaging, Diagnostic Department, Geneva University Hospitals, Geneva, Switzerland

**Keywords:** Biomarkers, Predictive markers, Biomarkers, Preclinical research, Molecular medicine

## Abstract

Assessment of amyloid deposits is a critical step for the identification of Alzheimer disease (AD) signature in asymptomatic elders. Whether the different amyloid processing methods impacts on the quality of clinico-radiological correlations is still unclear. We directly compared in 155 elderly controls with extensive neuropsychological testing at baseline and 4.5 years follow-up three approaches: (i) operator-dependent standard visual reading, (ii) operator-independent automatic SUVR with four different reference regions, and (iii) novel operator and region of reference-independent automatic Aβ-index. The coefficient of variance was used to examine inter-individual variability for each processing method. Using visually-established amyloid positivity as the gold standard, the area under the receiver operating characteristic curve (ROC) was computed. Linear regression models were used to assess the association between changes in continuous cognitive score and amyloid uptake values. In SUVR analyses, the coefficient of variance varied from 1.718 to 1.762 according to the area of reference and was of − 3.045 for the Aβ-index method. Compared to the visual rating, Aβ-index method showed the largest area under the ROC curve [0.9568 (95% CI 0.9252, 0.98833)]. The best cut-off score was of − 0.3359 with sensitivity and specificity values of 0.97 and 0.83, respectively. Only the Aß-index was related to more severe decrement of cognitive performances [regression coefficient: 9.103 (95% CI 1.148, 17.058)]. The Aβ-index is considered as preferred option in asymptomatic elders, since it is operator-independent, avoids the selection of reference area, is closer to established visual scoring and correlates with the evolution of cognitive performances.

## Introduction

Amyloid position emission tomography (PET) is a well-established imaging biomarker notably for the identification of preclinical Alzheimer Dementia (AD)^1^. Reproducing what was already described in human neuropathology, early PET studies showed that Aß deposits occur in multiple cortical areas (with the more frequent occurrence in frontal, precuneus and posterior cingulate cortex) preceding both tau accumulation and clinically overt dementia^2–5^. Compared with amyloid-negative, amyloid-positive controls showed moderate decline in verbal and visual episodic memory over 36 months^6^. Most importantly, the absence of amyloid in MCI cases is associated with cognitive stability at 36 months^7^. Moreover, changes in functional connectivity, brain volume, regional metabolism but also cognition occur even in cases with subthreshold amyloid deposition (defined as mean distribution volume ratio of amyloid lower than 1.07 or 1.3 independently of the visual inspection^8^. Since amyloid deposition is thought to precede synaptic dysfunction, increased cerebrospinal fluid (CSF) tau and phospho-tau levels, subtle structural changes in hippocampal volume and cortical thinning and ultimately cognitive impairment^9–12^, amyloid PET analysis tools should be tested in cognitively intact elderly persons.

A major limitation of amyloid PET is the variability of the results obtained with different processing paradigms (for review see Ref.^13^). To date, several data analysis techniques are available. The simplest analysis is a visual interpretation of the results. According to the Food and Drug Administration (FDA) and European Medicines Agency (EMA) documentation, this visual analysis is based on the notion that amyloid PET tracers physiologically bind only to the white matter (WM) yet not the grey matter (GM). If there is abnormal uptake in the GM in at least one anatomic region (parietal cortex, temporal cortex, frontal cortex, anterior cingulate cortex, posterior cingulate cortex/precuneus and striatum) the scan is considered abnormal. While this visual analysis does not need any specific post-processing tools, it requires time from an experienced human reader, is operator-dependent, and thus be prone to intra- and inter-rater discrepancies. Another operator-independent analysis method is automatic spatial normalization of the individual brain into an atlas standard space (oftentimes the Montreal neurological institute MNI space) followed by the automatic assessment of standardized uptake value (SUV) ratio (SUVR) between a set of predefined atlas regions versus a reference region. SUVR cut-off values are specific for each tracer^14^. If different PET tracers are used, the Centiloid processing^15^ might be applied to overcome the problem of tracer-specific reference values by scaling the available amyloid PET tracers relative to the PiB-PET ([C-11] Pittsburgh Compound-B positron emission tomography) as reference standard. Although less exposed to inter-rater variability^16,17^, the resulting SUVR values vary depending on the selected reference region, i.e. central pons, thalamus-pons, cerebellar cortex and whole cerebellum and brain stem. The concordance between quantitative and visual real categorization was close to 0.95 in clinically overt AD cases^18^. However, this value reached only 0.74 in MCI cases, the visual analysis having lower sensitivity but better specificity compared to quantitative SUVR data^19^. Asymptomatic elderly cases with low amyloid burden would represent one among the possible targets for future curative treatments. When moving to these less affected cases, the performance of currently available tools for amyloid assessment becomes questionable. In this population, quantitative SUVR threshold may be a good screening tool for detecting early amyloid deposits in cases with visual negative reads, but not specific enough to make clinical inferences^20^. Moreover and in the absence of longitudinal follow-up of cognitive performances, it is difficult to establish the relevance of these methods in the prediction of the very initial phases of cognitive decrement. In fact, low levels of amyloid load per se is thought to be associated with a less than 10% increase of lifetime risk for AD in the oldest-old but more than 25% increase in younger elderly persons^21^.

More recently developed methods rely on user- and region-independent strategies for the assessment of amyloid load^22^. In particular, the Aβ-index derived from the method described by Lilja et al.^23^ combines the advantages of an automatic, user-independent data processing with an independence of the selection of a reference region. This method calculates an Aβ-index using the weighting factor measured during spatial normalization utilizing an adaptive principal component template. We had the opportunity to explore the performance of this method compared to visual inspection and quantitative SUVR data with various areas of reference in a large community-based cohort of cognitively preserved elders with full neuropsychological documentation at baseline and 4.5 years cognitive follow-up.

## Materials and methods

The study population has been described and published previously, for example Refs.^24,25^.

### Study population

The study was performed in agreement with the declaration of Helsinki and approved by the ethical committee of Geneva, Switzerland. All participants gave written informed consent. All of the cases were recruited via advertisements in local newspapers and media. The cohort included 526 elderly Caucasian white individuals living in Geneva and Lausanne catchment area. Due to the need for an excellent French knowledge (in order to participate in detailed neuropsychological testing) the vast majority of the cohort were Swiss (or born in French-speaking European countries, 92%)^26–28^. Education was assessed as an ordinal variable as a function of the formal years of training (< 9: obligatory, 9–12: high school, > 12: university). We included in the current investigation those individuals who met the following inclusion criteria: (i) three neurocognitive assessments (see below) at baseline, 18 months and 54 months, (ii) structural brain MRI at baseline and 54 months, (iii) brain amyloid PET and (iv) APOE status at baseline. Our sample included 155 elderly individuals (mean age at inclusion 71.8 ± 4.3, range 64–86 years, 96 (61.9%) females; Table 1).

**Table 1:** Clinical and demographic data in the present series.

	Gender		Total	*p* Value
	Female	Male		
*N*	96	59	155	
Age at Amy PET	77.9 ± 4.2	78.2 ± 4.4	78.0 ± 4.3	0.680
**Education (year)**				< 0.01
< 9	22 (22.9%)	1 (1.7%)	23 (14.8%)	
9–12	45 (46.9%)	26 (44.1%)	71 (45.8%)	
> 12	29 (30.2%)	32 (54.2%)	61 (39.4%)	
MMSE at baseline	28.5 ± 1.4	28.5 ± 1.6	28.5 ± 1.5	0.994
**APOE4**				0.663
Negative	84 (87.5%)	53 (89.8%)	137 (88.4%)	
Positive	12 (12.5%)	6 (10.2%)	18 (11.6%)	
**Visual Score Amy PET**^a^				0.775
Negative	67 (69.8%)	40 (67.8%)	107 (69.0%)	
Positive	29 (30.2%)	19 (32.2%)	48 (31.0%)	

^a^Seventy-seven ^18^F-Florbetapir- (Amyvid) and seventy-eight ^18^F-Flutemetamol-PET (Vizamyl).

### Neurocognitive assessment

At baseline, all individuals were evaluated with an extensive neuropsychological battery, including the Mini-Mental State Examination (MMSE)^29^, the Hospital Anxiety and Depression Scale (HAD^30^, and the Lawton Instrumental Activities of Daily Living (IADL^31^). Cognitive assessment included (a) attention (Digit-Symbol-Coding^32^, Trail Making Test A^33^, (b) working memory (verbal: Digit Span Forward^34^), visuo-spatial: Visual Memory Span (Corsi)^35^, (c) episodic memory (verbal: RI-48 Cued Recall Test^36^), visual: Shapes Test ^37^, (d) executive functions (Trail Making Test B^33^, Wisconsin Card Sorting Test and Phonemic Verbal Fluency Test^38^, (e) language (Boston Naming^39^, (f) visual gnosis (Ghent Overlapping Figures), (g) praxis: ideomotor^40^, reflexive^41^, and constructional (Consortium to Establish a Registry for Alzheimer’s Disease (CERAD), Figures copy^42^). All individuals were also evaluated with the Clinical Dementia Rating scale (CDR)^43^. In agreement with the criteria of Petersen et al.^44^, participants with a CDR of 0.5 but no dementia and a score exceeding 1.5 standard deviations below the age-appropriate mean in any of the cognitive tests were classified as MCI and were excluded. Participants with neither dementia nor MCI were classified as cognitively healthy controls and underwent full neuropsychological assessment at follow-ups, on average 18 and 54 months later. The subtle cognitive decline was defined with a continuous cognitive score (CCS) computed as follows. Most of the cognitive performances, discrete or continuous, cannot be linearly combined by adding the individual scores to a unique composite cognitive score. Thus, all values were converted to z scores. Subsequently, we summed the number of cognitive tests at follow-up with performances at least 0.5 standard deviation (SD) higher compared with the first evaluation, leading to the number of tests with improved performances (range 0–14). Similarly, we summed the number of cognitive tests at follow-up with performances at least 0.5 SDs lower compared with the first evaluation, yielding the number of tests with decreased performances (range 0–14). Finally, the number of tests with improved minus the number of tests with decreased performances results in a final CCS. Change in cognition between inclusion and last follow-up was defined as the sum of the continuous cognitive scores at two follow-ups.

### APOE epsilon 4 status

APOE epsilon 4 status was assessed as described earlier^28^. Subjects were divided according to whether they were a carrier of the APOE epsilon 4 allele (4/3 versus 3/3, 3/2 carriers).

### Amyloid PET imaging

Seventy-seven ^18^F-Florbetapir- (AMYVID) and seventy-eight ^18^F-Flutemetamol-PET (VIZAMYL) data were acquired on 2 different tomographs (SIEMENS Biograph mCT and GE Healthcare Discovery PET/CT 710 scanners) of varying resolution and following different platform-specific acquisition protocols (for details see Refs.^24,25^). The ^18^F-Florbetapir images were acquired 50–70 min after injection and the ^18^F-Flutemetanol images 90–120 min after injection. PET images were reconstructed using the parameters recommended by the ADNI protocol aimed at increasing data uniformity across the multicenter acquisitions. More information on the different imaging protocols for PET acquisition can be found on the ADNI web site (https://adni.loni.usc.edu/wp-content/uploads/2012/10/ADNI3_PET-Tech-Manual_V2.0_20161206.pdf).

### Visual amyloid PET analysis

The visual analysis of amyloid PET images was conducted by an independent, board-certified specialist in nuclear medicine (VG), following the tracer-specific standardized operating procedures approved by the European Medicinal Agency. Specifically, regional positivity was assessed for each scan, specifying if uptake was identified in the frontal lateral, parietal lateral, posterior cingulate and precuneus, anterior cingulate, temporal lateral and striatal regions in either of the two hemispheres. Since visual rating was used as gold standard in further analyses, two independent readers following the same instructions classified all of the cases with high inter-rater reliability (kappa = 0.90). The classification of discordant cases was made by a senior radiologist blind to the previous readings.

### Automatic spatial normalization and Aβ-index

Images were spatially normalized to MNI space using a PET driven image registration utilizing the adaptive principal component template method described by Lilja et al.^23^. The complete details of the principal component approach can be found in the original publication. Briefly, the adaptive template is created based on tracer specific principal component images calculated by singular value decomposition. A synthetic template, *I*_*Synthetic*_, can then be modelled as a linear combination of the first principal component image, *I*_*PC1*_, and the second principal component image, *I*_*PC2*_, i.e.

$$ I_{Synthetic} = I_{PC1} + A\beta { - }index \, \times \, I_{PC2} , $$

where a positive *Aβ-index* yields a template with a more Aβ-positive appearance and a negative *Aβ-index* yields a template with a more Aβ-negative appearance. The spatial normalization optimizes both spatial parameters and the *Aβ-index* when registering images to the adaptive template defined in MNI space. In the present study pre-existing synthetic templates derived from clinical trial phase 2 studies were used for spatial normalization of ^18^F-Florbetapir^45,46^ and ^18^F-Flutemetamol^47^. The values for Aß-index were averaged across the two tracers. The registration method is similar to that reported by Lundqvist et al.^48^. The difference lies in the creation of the adaptive template. In the implementation of CortexID, these authors use a linear regression model whereas the method described by Lilja et al.^23^ utilizes eigenvalue decomposition. Visual comparison of resulting templates suggests greater accuracy with the latter method.

### Cortical SUVR analysis

Cortical SUVR values were calculated using reference regions as provided by the centiloid project^15^; pons, cerebellum grey matter, whole cerebellum and whole cerebellum + brain stem. Cortical uptake was calculated using the regions from the Harvard–Oxford atlas^49^.

### Statistical analysis

The Pearson’s correlation coefficients were used to assess the strength of the association between two amyloid processing methods. The coefficients of variation were computed as 100 × SD/mean. The Pitman's Test of difference in variance was used to examine the concordance between the quantitative amyloid-assessing methods. Using the binary amyloid positivity as the gold standard, the area under the receiver operating characteristic curve (ROC) was computed along with its binomial exact 95% confidence interval for each quantitative amyloid-assessing method using the native values and their z-scores. Chi-square was used to compare the values of the area under the ROC curves between them. We also perform additional analysis building logistic regression models to explore the ability of the z-scores of amyloid processing methods in the discrimination between visually assessed amyloid positive and negative cases. Linear regression models controlling for age, gender, education and APOE genotype were used to assess the association between changes in continuous cognitive score and amyloid values according to the different methods of assessment. A threshold of p value less than 0.05 was applied for significance. The “Stata” software release 16.0 was used for all analysis.

### Statement of ethics

The study was approved by the local ethical committee and all participants gave written informed consent.

## Results

Table 1 summarizes the demographic, clinical and main amyloid PET imaging data according to the different technics used. The mean MMSE score was of 28.49 at the second follow-up whereas a slight decrement of the continuous cognitive score was observed between inclusion and second follow-up (mean value of − 0.78). Only 31% of cases were amyloid-positive in binary visual inspection.

Correlation coefficients illustrate the association between two amyloid processing methods. The automatic Aß-index was significantly associated with all SUVRs (SUVR pons, r = 0.611, p = 0.0001, SUVR cerebellar gray matter r = 0.869 SUVR whole cerebellum, r = 0.906, p = 0.0001, SUVR whole cerebellum + brain stem, r = 0.875, p = 0.0001). Strong associations were also found between all four SUVR methods (r values ranging from 0.408 between cerebellar gray matter and pons to 0.982 between whole cerebellum and whole cerebellum + brain stem, p = 0.0001).

The mean values of amyloid scores according to the different methods are summarized in Table 2. There were striking differences in the inter-individual variability between the amyloid-assessing methods. Concerning the automatic SUVR analysis, the coefficient of variation was of 1.718 for the cerebellar gray matter as reference region, 1.762 for the pons, 1.727 for the whole cerebellum and 1.740 for the whole cerebellum + brain stem. The automatic Aβ-index method showed a coefficient of variance of -3.045. There were significant differences in variance between the automatic Aβ-index and SUVR pons (r = 0.696, p = 0.0001), SUVR whole cerebellum (r = 0.724, p = 0.0001) as well as SUVR whole cerebellum + brain stem (r = 0.752, p = 0.0001). However, this was also the case between the SUVR methods depending on the region of reference [cerebellar gray matter higher than the three other regions, r values 0.613, 0.656, 0.686 respectively, p = 0.0001; pons lower than whole cerebellum (r = − 0.381, p = 0.001)], and whole cerebellum + brain stem (r = − 0.326, p = 0.0001).

**Table 2:** Average values [along with standard deviation (SD), minimum, maximum and coefficient of variation (CV)] obtained for amyloid PET according to the processing method (N = 155).

Region	Mean	SD	Min	Max	CV	(z-score difference)	95% CI		OR	95% CI		AUC	95% CI	
Automatic Aβ-index	− 0.38	0.01	− 0.40	− 0.37	− 3.04	− 1.69	− 1.96	− 1.41	51.9	12.8	210.5	0.96	0.93	0.99
Cerebellum gray matter	1.24	0.02	1.22	1.27	1.72	− 1.55	− 1.85	− 1.24	30.5	9.4	98.6	0.94	0.90	0.98
Pons	0.59	0.01	0.58	0.60	1.76	− 0.90	− 1.25	− 0.55	2.7	1.8	4.1	0.74	0.65	0.82
Whole cerebellum	0.97	0.02	0.96	0.99	1.73	− 1.53	− 1.85	− 1.20	19.5	7.1	53.3	0.92	0.87	0.97
Whole cerebellum + brain stem	0.88	0.02	0.87	0.90	1.74	− 1.44	− 1.77	− 1.11	10.0	4.6	21.6	0.88	0.82	0.94

The discrimination between visually assessed amyloid positive and negative cases was assessed by the magnitude of separation computed as the difference between z-scores, the odds ratio (OR) computed by logistic regression and area under the ROC curves (AUC). All p values < 0.0001.

Logistic regression analysis and areas under the ROC curve (AUC) using the z-scores of amyloid processing methods that the automatic Aβ-index yielded the best OR and AUC compared to the four SUVR analyses (Table 2). Representative Bland–Altman plots are provided in Supplementary Fig. 1.

**Figure 1:**
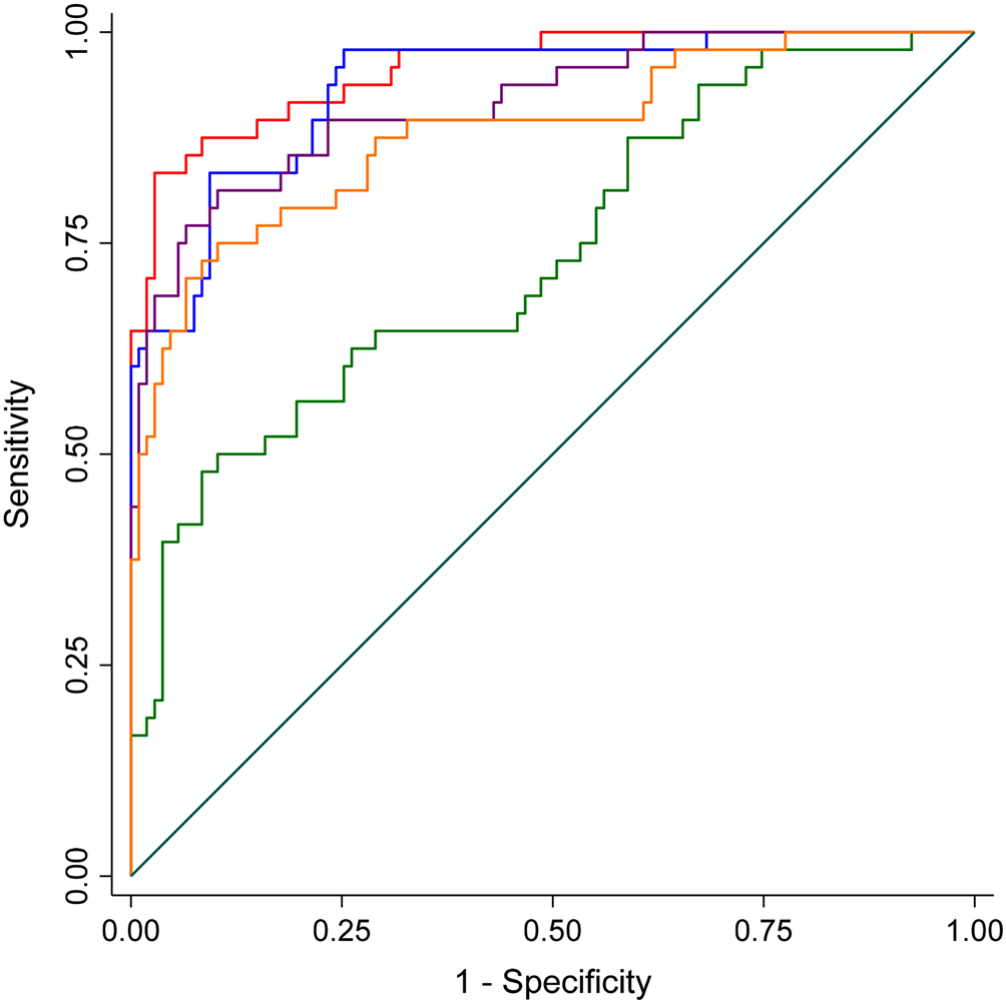
ROC curves predicting the global visual score (gold standard) from automatic Aß-index method or cortical standard uptake value ratios (SUVR). Aß-index (red); cerebellum gray matter (blue), pons (green), whole cerebellum (purple), whole cerebellum + brain stem (orange) SUVR.

In addition, the areas under the ROC curve using the native values for each quantitative amyloid-assessing method (with the visually established amyloid positivity as gold standard) are illustrated in Fig. 1. The automatic Aβ-index showed the largest area under the ROC curve [0.9568 (95% CI 0.9252, 0.98833)] followed by SUVR cerebellar gray matter [0.9356 (95% CI 0.89564, 0.97546]. All of the other methods displayed significantly smaller areas under the ROC curve. The difference between the automatic and the various SUVR regions was significant for pons (Chi square = 33.31, p = 0.0001), whole cerebellum (Chi square = 7.31, p = 0.007) and whole cerebellum + brain stem (Chi square = 12.82, p = 0.0003). A best cut-off score of − 0.339 was defined with sensitivity and specificity values of 0.97 and 0.83, respectively.

We also explored the association between the different amyloid-assessing methods and decrement of cognitive performances in the present series. There were no significant differences in CCS between amyloid positive [n = 48, − 1–33 (− 2.45, − 0.21)] and amyloid negative [n = 107, − 0.53 (− 1.17, 0.11)] (p = 0.28). Supporting the validity of the CCS, the cognitive decrement is significantly different from zero for the 48 amyloid positive cases whereas this was not the case among amyloid negative cases. Mean SUVR values according to the areas of reference were all unrelated to the cognitive outcome in this cohort of cognitively normal individuals (cerebellar gray matter: − 1.65, p = 0.217, pons: − 1.68, p = 0.16, cerebellum: − 2.71, p = 0.168, whole cerebellum + brain stem: − 2.76, p = 0.201). In contrast, higher Aβ-index score was related to more severe decrement of cognitive performances (decrease of the continuous cognitive score at the second follow-up compared to baseline) [regression coefficient: 9.103 (95% CI 1.148, 17.058), p = 0.026].

## Discussion

The present data reveal that the type of amyloid processing has a significant impact on inter-individual variability and clinico-radiological associations in asymptomatic elderly individuals. Most importantly, they indicate that an automatic, user- and region of reference-independent Aß-index is the only among the methods tested to be associated with subtle changes in cognitive performances in normal aging.

There was a substantial inter-individual variability in PET amyloid uptake independently of the processing used. At first glance, this finding is not surprising given the well-known inter-individual variability of amyloid accumulation in florbetapir-based amyloid imaging in elderly controls^50^. However, we also observed a significant difference in the inter-individual variability across the different processing methods. The SUVR approach with pons as reference region and automatic Aß index displayed the lower and higher inter-individual variability respectively. Most importantly, significant inter-individual variability differences were observed between the different SUVR techniques depending on the area of reference. These data indicate that both variability of tracers (flutemetamol and florbetapir compared to PET-PiB^50^) and different processing methods may heavily impact on inter-individual variability in a given cohort. In order to avoid erroneous estimations of amyloid rate increase, it is thus crucial to keep the experimental design strictly identical across the different time points not only for tracers but also processing methods.

In the absence of autopsy validation, we used the visual rating as reference standard since this technique is widely used in clinical routine and the corresponding standardized procedure has been approved by the European Medicinal Agency. Our results show that among the different amyloid processing techniques, the Aβ-index was the more closely related to the visual score, significantly closer than the different SUVR measures with the exception of that with reference to the cerebellar gray matter. Importantly, the sensitivity and specificity values for predicting visual reading with a best cut-off Aß-index score of − 0.339 were excellent (0.97 and 0.83, respectively). Previous studies showed that the concordance between visual rating and SUVR measures is extremely high in AD, but decreases progressively when moving to MCI and cognitively intact cases^18–20^. In cases with low amyloid load, similar to those included in our series, the use of SUVR values may lead to an overestimation of cortical amyloid burden^51^. Our findings are close to those reported by Chincarini et al.^22^ using a semi-quantitative approach (ELBA) based on the evaluation of the whole brain without specific regions of interest. However, this method was operator-dependent and no data are available on its association with cognitive decrement over time.

In the present cohort, only the Aβ-index had a significant positive correlation with cognitive changes over a 4.5 year follow-up period. The visual rating and the various SUVR approaches did not correlate with the decrement of cognitive performances in our cohort. Previous studies showed that SUVR measures in ^18^F-Flutemetamol-PET amyloid scans have a specificity of 80% and specificity of 89% in predicting MCI transition to AD. These values were much lower for visual rating. For ^18^F-Florbetapir amyloid scans, the specificity values were very low (50–52%) for both amyloid processing methods^52,53^. In healthy controls, the use of SUVR measures and visual rating to predict decreased memory performances led to conflicting data. Cross-sectional studies failed to identify solid associations between ^18^F-Flutemetamol or Florbetapir data (both visual cut-off and quantitative) and cognitive decrement^54,55^. Longitudinally, the INSIGHT-preAD study demonstrated that ^18^F-Florbetapir SUVR values were not related to significant changes in MMSE scores over a 30-month period^56^. For some authors, amyloid deposition can still have a direct deleterious impact on cognition that remains independent on tau accumulation and neurodegeneration^57^. Our results show that the amyloid processing may also impact on the quality of clinic-radiologic correlations in cognitively normal cases with low amyloid burden at baseline. In this context, the Aß index is the only to reveal a deleterious impact of amyloid on the subtle changes in cognitive score. However, one should keep in mind that our data concerned only mean SUVR values that has been shown to underperform in the prediction of cognitive decline in healthy controls compared to regional SUVR. In fact, accumulation of amyloid in posterior cortical areas measured by SUVR may predict the decline in episodic memory in initially amyloid-negative adults^58^. In this perspective, Aß index could be a rapid and unbiased alternative to visual reading for less experienced readers that, in terms of cognitive trajectories, could be completed by the analysis of amyloid load in posterior cortical areas.

Strengths of the present study include the exclusion of cases with significant vascular burden affecting the cognitive performances, careful neuropsychological assessment over the 4.5-year follow-up period, and use of both binary and continuous assessments of amyloid burden. Two main limitations should also be noted. First, in the absence of autopsy validation, no biologically relevant gold standard was available limiting the understanding of the results in relation to underlying AD pathology. Second, though tracer specific templates were used for the two cohorts in the study, it remains to be determined in future studies how the absolute values of Aß-index differ across tracers. Future studies including longitudinal assessment of cognition and autopsy validation of amyloid distribution are needed to further explore the relevance of Aß-index as a new measure of amyloid burden in normal aging.

## Supplementary information

Supplementary Information.

## Abbreviations

AD: Alzheimer dementia
APOE: Apolipoprotein E
MCI: Mild cognitive impairment
PET: Positron emission tomography
