# Supplementary material for: PET amyloid in normal aging: direct comparison of visual and automatic processing methods
Source: Sci Rep. 2020 Oct 7;10:16665. doi: 10.1038/s41598-020-73673-1 (PMC7542434; doi:10.1038/s41598-020-73673-1)
Supplement: Supplementary file 1 — Supplementary Information. [file 41598_2020_73673_MOESM1_ESM.docx]

**Research Article**

**Title**

PET amyloid in normal aging: direct comparison of visual and automatic processing methods

**Short Title:**

Amyloid PET analysis methods

**Authors**

Sven HALLER a,b,c

Marie-Louise MONTANDON d,e

Johan LILJA b, f, g

Cristelle RODRIGUEZ e,h

Valentina GARIBOTTO c,i,

François R. HERRMANN d

Panteleimon GIANNAKOPOULOS e,h

**Affiliations**

a CIRD Centre d’imagerie Rive Droite, Geneva, Switzerland

b Department of Surgical Sciences, Radiology, Uppsala University, Sweden

c Faculty of Medicine, University of Geneva, Switzerland

d Department of Rehabilitation and Geriatrics, Geneva University Hospitals and University of Geneva, Switzerland

e Department of Psychiatry, University of Geneva, Switzerland

f Clinical Memory Research Unit, Department of Clinical Sciences, Lund University, Malmö, Sweden

g Hermes Medical Solutions, Stockholm, Sweden

h Division of Institutional Measures, Medical Direction, Geneva University Hospitals, Switzerland

i Division of Nuclear Medicine and Molecular Imaging, Diagnostic Department, Geneva University Hospitals, Switzerland

**Corresponding Author**

Prof. Dr. M.Sc. Sven Haller

1 CIRD Centre d’imagerie Rive Droite, Geneva, Switzerland

2 Department of Surgical Sciences, Radiology, Uppsala University, Uppsala, Sweden

3 Faculty of Medicine of the University of Geneva, Switzerland

sven.haller@me.com

**Keywords:**

aging, hippocampus, amyloid, PET

**Supplementary Figure 1**

Supplementary Figure 1: Representative Bland-Altman plots for the automatic Aβ-index in different brain reference regions.
